# Metabolic pathways of eicosanoids—derivatives of arachidonic acid and their significance in skin

**DOI:** 10.1186/s11658-025-00685-y

**Published:** 2025-01-17

**Authors:** Michał Biernacki, Elżbieta Skrzydlewska

**Affiliations:** Department of Analytical Chemistry, Medical University of Bialystok, Kilinskiego 1, 15-069 Bialystok, Poland

**Keywords:** Eicosanoids, Phospholipids metabolism, Phospholipases, Fatty acids, Cyclooxygenases, Lipoxygenases, Cytochrome P450, Prostaglandins, Leukotrienes

## Abstract

The skin is a barrier that protects the human body against environmental factors (physical, including solar radiation, chemicals, and pathogens). The integrity and, consequently, the effective metabolic activity of skin cells is ensured by the cell membrane, the important structural and metabolic elements of which are phospholipids. Phospholipids are subject to continuous transformation, including enzymatic hydrolysis (with the participation of phospholipases A, C, and D) to free polyunsaturated fatty acids (PUFAs), which under the influence of cyclooxygenases (COX1/2), lipoxygenases (LOXs), and cytochrome P450 (CYPs P450) are metabolized to various classes of oxylipins, depending on the type of PUFA being metabolized and the enzyme acting. The most frequently analyzed oxylipins, especially in skin cells, are eicosanoids, which are derivatives of arachidonic acid (AA). Their level depends on both environmental factors and endogenous metabolic disorders. However, they play an important role in homeostasis mechanisms related to the structural and functional integrity of the skin, including maintaining redox balance, as well as regulating inflammatory processes arising in response to endogenous and exogenous factors reaching skin cells. Therefore, it is believed that dysregulation of eicosanoid levels may contribute to the development of skin diseases, such as psoriasis or atopic dermatitis, which in turn suggests that targeted control of the generation of specific eicosanoids may have diagnostic significance and beneficial therapeutic effects. This review is the first systemic and very detailed approach presenting both the causes and consequences of changes in phospholipid metabolism leading to the generation of eicosanoids, changes in the level of which result in specific metabolic disorders in skin cells leading to the development of various diseases. At the same time, existing literature data indicate that further detailed research is necessary to understand a clear relationship between changes in the level of specific eicosanoids and the pathomechanisms of specific skin diseases, as well as to develop an effective diagnostic and therapeutic approach.

## Introduction

The skin, acting as a barrier between the human body and the external environment, protects the body from mechanical injuries and the effects of external factors, such as physical [solar radiation, including ultraviolet (UV), temperature, and wind] and chemical (e.g., disinfectants, detergents) factors, as well as pathogens (bacteria, viruses, etc.) [1]. Additionally, it performs secretory and excretory functions [2]. Skin is also responsible for the biosynthesis of many hormones and hormone-like substances, including corticosteroids, androgens, and estrogens [3, 4], e.g., the biosynthesis of cholecalciferol (vitamin D_3_) and its active metabolites [5]. Furthermore, skin is involved in the metabolism of glucose, proteins, and lipids [6, 7].

The skin has a multilayered structure consisting of the epidermis, dermis, and subcutaneous tissue, where keratinocytes are the dominant epidermal cells. In the basal layer of the epidermis there are melanocytes that produce pigment and perform protective functions [8] and Langerhans cells, which, among others, process microbial antigens, playing a key role in immune mechanisms of the skin [9]. However, in the dermis, fibroblasts are surrounded by an extracellular matrix composed of glycosaminoglycans, proteoglycans, and structural proteins, such as collagen and elastin, as well as macromolecules, fibrin, and hyaluronic acid, which ensure skin elasticity and mechanical resistance [10]. Blood and lymphatic vessels are also located in this part of the skin, supplying oxygen and performing nutritional functions, as well as providing a transport route for immune cells [11].

The integrity and, consequently, the effective metabolic activity of individual skin cells is ensured by the cell membrane, which separates the cell’s interior from its environment and regulates transport and signaling between skin cells and their surroundings [7, 12]. The cell membranes of epidermal and dermal cells contain various types of lipids, including sterols, phospholipids, ceramides, and glycosphingolipids [13]. Among the structural components of the cell membrane, a metabolically important group are phospholipids [12, 13], which are found in the largest amounts in the cells of the basal layer, including keratinocytes, where they constitute about 70% of all cellular lipids [12]. The stability of the lipid bilayer structures of membranes depends on the composition of phospholipids as well as lipid-lipid and lipid-integral membrane proteins interactions [14, 15]. The functions of proteins and other membrane biomolecules are influenced by cholesterol, which, by modifying the rotation and diffusion of phospholipids, ensures the appropriate fluidity of the cell membrane [16, 17]. In addition to providing physiological conditions, membrane phospholipids also act as a barrier against environmental microorganisms by inhibiting bacterial cell membrane biosynthesis and modifying their metabolism [18].

Animal studies have shown that UV radiation contained in sunlight (UVA and UVB) modifies the level of various classes of lipids in animal skin keratinocytes (in vivo) [19], as well as in human fibroblasts irradiated in vitro [20]. Moreover, the development of skin diseases, such as psoriasis and atopic dermatitis, is accompanied by disorders of phospholipid metabolism in skin cells [21, 22].

In psoriasis, T lymphocytes are activated, which results in hyperproliferation of keratinocytes leading to increased production of free arachidonic acid and, consequently, proinflammatory mediators from the eicosanoid group [23]. This is accompanied by reduced levels of free short-chain fatty acids and increased cholesterol levels [24] as well as dysregulation of ceramide levels along with changes in their stratum corneum subtypes [25]. However, it should be noted that extracellular ceramides are necessary to maintain the skin’s water-holding capacity and permeability barrier, while intracellular ceramides play a key role in promoting keratinocyte differentiation [26]. One of the consequences of dysregulation of ceramide levels is damage to the epidermis, disruption of the epidermal barrier and, consequently, increased transepidermal water loss [27]. Moreover, it has been shown that the reduction of ceramide synthesis and their level in the epidermis positively correlates with the Psoriasis Area and Severity Index (PASI) in psoriasis [28]. Moreover, changes in the level of most groups of skin cell lipids were also found in keratinocytes isolated from patients with psoriasis [29].

However, in atopic dermatitis (AD), a decrease in the level of total lipids and dysregulation of the level of individual lipids is observed, as well as a decrease in the level of ceramides and a decrease in the length of the fatty acid chains of stratum corneum ceramides [12], which is accompanied by the accumulation of phospholipids [30]. It has also been found that oxidative stress accompanying the development of atopic dermatitis leads to increased trans isomerization of polyunsaturated fatty acids (PUFA) in membrane lipids, which contributes to increased lipid peroxidation, which disturbs the physiology of biological membranes and intracellular metabolism [31]. Moreover, in AD, there is an increased generation of lipid mediators, including arachidonic acid derivatives, which favors the modification of inflammation [32].

## Skin lipids and their metabolism

Skin lipids are primarily produced by keratinocytes, sebocytes, and the skin microbiome [33]. Moreover sebocytes generate substances, such as squalene, triglycerides, fatty acids, wax esters, cholesterol, and cholesterol esters, which are secreted onto the skin’s surface. In keratinocytes, the synthesis of lipid precursors for the stratum corneum takes place, which, through the action of lipid synthases, leads to the formation of lipids in the stratum corneum, mainly ceramides, fatty acids, and cholesterol [34].

Lipid analysis of epidermal and dermal cell membranes showed that they contain twelve types of these compounds, including phospholipids, such as phosphatidic acid (PA), phosphatidylethanolamines (PE), cardiolipins (CL), phosphatidylserine (PS), lysophosphatidylcholines (LPC), phosphatidylinositols (PI), alkylacylglycerophosphocholines (AAPC), plasmalogen ethanolamines (Epla), and phosphatidylcholines (PC), as well as sphingolipids (SP), such as dihydrosphingomyelins (DHSM), sphingomyelins (SM), and ceramides (Cer) [13, 35]. The lipids forming the skin’s lamellar barrier consist of 50% ceramides in the stratum corneum, primarily CER[AH], CER[NS] and CER[AS] [36]. The source of lipids is also the skin microbiome, whose metabolism is based mainly on short-chain fatty acids [1]. Furthermore, lipids, mainly ceramides, are also synthesized in other cells such as fibroblasts and melanocytes, though in much smaller quantities [37, 38]. Among them, PCs are found in the largest amounts and higher levels of PEs are observed in the deeper layers of the epidermis, while in the stratum corneum ceramides are found in greater amounts [13].

It is known that the metabolic stability of skin cells is largely dependent on the phospholipid composition of the bilayer of their cell membranes [12, 13]. However, the structure and level of individual phospholipids in biological membranes depend on both the biosynthesis and metabolism of these compounds. In the process of phospholipid biosynthesis in mammalian cells, which is a complex and multidirectional process, phosphatidic acid plays a key role [12, 39].

**Fig. 1:**
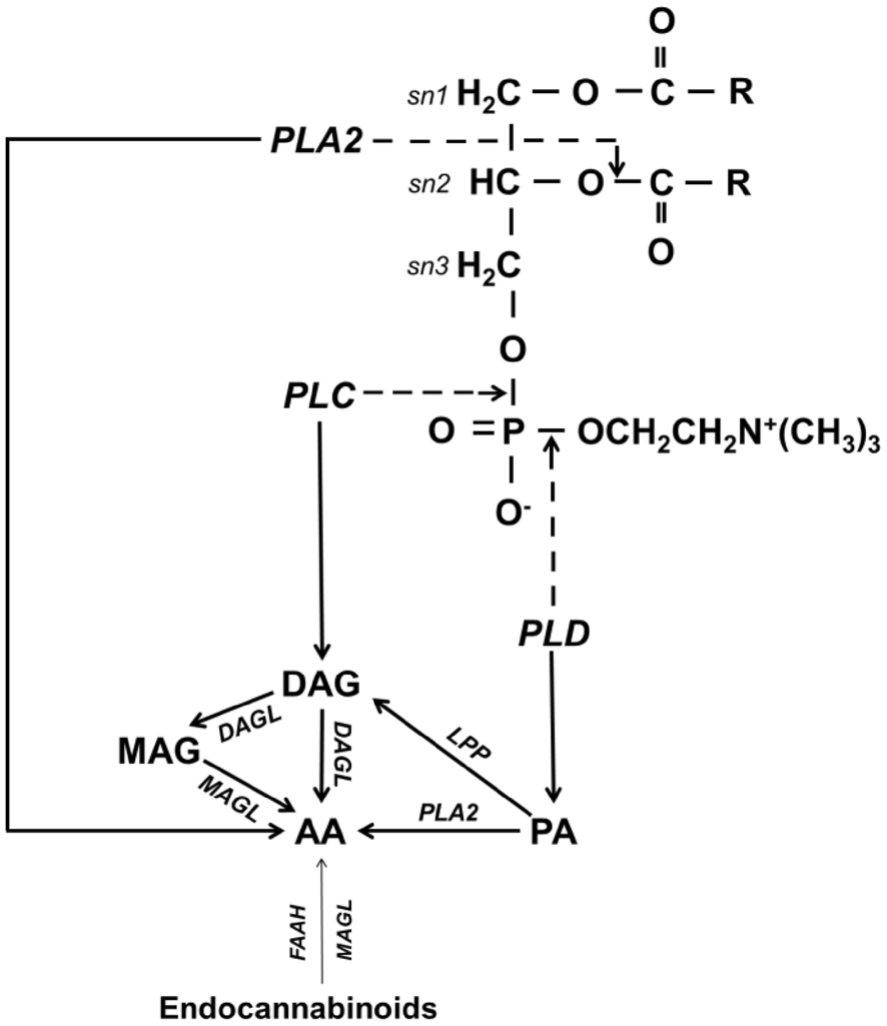
Metabolism of membrane phospholipids and endocannabinoids with the release of arachidonic acid. *AA* arachidonic acid, *DAG* diacylglycerol, *DAGL* diacylglycerol lipase, *FAAH* fatty acid amide hydrolase, *LPP* lipid phosphate phosphatase, *MAG* monoacylglycerol, *MAGL* monoacylglycerol lipase, *PA* phosphatidic acid, *PLA2* phospholipase A2, *PLC* phospholipase C, *PLD* phospholipase D

As a result of oxidation processes intensified by external factors (UV radiation, chemical factors, pathogens), there is an overproduction of reactive oxygen species (ROS) in skin, which is usually accompanied by a reduction in antioxidant capacity, which intensifies, among others, oxidative metabolism of phospholipids to produce lipid peroxidation products, such as small unsaturated aldehydes, including 4-hydroxynonenal (4-HNE) and malondialdehyde (MDA), as well as cyclic fatty acid derivatives [isoprostanes (IsoP), isothromboxanes (IsoTX), isolevuglandins (IsoLG), and isofurans (IsoFS)] [40, 41]. However, phospholipid metabolism is primarily associated with their enzymatic hydrolysis involving phospholipases, leading to the release of free fatty acids, including polyunsaturated fatty acids (PUFAs), and their enzymatic metabolism generating reactive products, such as eicosanoids and endocannabinoids [42–44]. As a result of the hydrolysis of phospholipid ester bonds by phospholipases, bioactive metabolites are generated, which not only affect cellular metabolism but also cause continuous remodeling of cell membranes [44, 45], thereby ensuring their proper functioning adapted to the cell’s requirements [42, 45]. Consequently, it is believed that modifications in the activity of phospholipases controlling the level of membrane phospholipids may constitute a component of skin disease therapy [42] (Fig. 1).

In skin, there are enzymes belonging to four main classes of phospholipases, designated as: A (A1, A2), B, C, and D, among which phospholipases A1/2 and B release fatty acids, while phospholipases C and D act as phosphodiesterases, participating in the generation of phosphatidic acid and 1,2-diacylglycerol (DAG), which undergo metabolism into free fatty acids [46]. Phospholipase A1 (PLA1) catalyzes the hydrolysis of the ester bond at the sn-1 position, where saturated fatty acids are located; whereas phospholipase A2 (PLA2) catalyzes the hydrolysis of the ester bond at the sn-2 position of glycerophospholipids, mainly phosphatidylcholine and phosphatidylethanolamine, usually leading to the release of polyunsaturated fatty acids, including arachidonic acid (AA) [47–49], while phospholipase B (PLB) hydrolyzes acyl chains from both the sn-1 and sn-2 positions; however, the full catalytic mechanism of phospholipase B is not yet fully understood [50]. However, phospholipase C (PLC) catalyzes the hydrolysis of 4,5-bisphosphate phosphatidylinositol (PIP2) to generate inositol-1,4,5-triphosphate (IP3) and DAG [51], which, under the action of diacylglycerol lipase (DAGL), undergoes metabolism into AA and monoacylglycerol (MAG), which then, under the action of monoacylglycerol lipase (MAGL), is hydrolyzed into glycerol and free AA [44]. Phospholipase D (PLD) is responsible for hydrolyzing the phosphodiester bond of phosphatidylcholine, releasing choline and PA—a glycerophospholipid, which under the action of PLA2 is then hydrolyzed into free arachidonic acid and lysophosphatidic acid (LPA). Phosphatidic acid is metabolized by lipid-phosphate phosphatase (LPP) to DAG, and further, as shown above, to arachidonic acid [52]. Additionally, endocannabinoids and their derivatives, belonging to the group of ester, ether, or amide derivatives of fatty acids, are hydrolyzed by fatty acid amide hydrolase (FAAH) to ethanolamine and fatty acid, and by MAGL to glycerol and fatty acid [43]. Owing to the low concentration of endocannabinoids in the skin, the level of free fatty acids arising from endocannabinoids is several orders of magnitude lower than those arising from phospholipids [53].

## Skin phospholipases

### PLA2

The primary phospholipase found in skin is PLA2, with the mammalian genome encoding over 50 isoforms of PLA2 or related enzymes, which are divided into several families on the basis of their structure and function. There are three main families of phospholipase A2: secretory—(sPLA2), cytosolic calcium dependent (cPLA2), and cytosolic calcium independent (iPLA2). The cPLA2 family comprises six isoforms (α-ζ), while the iPLA2 family consists of nine isoforms. Isoenzymes from the iPLA2 family are designated as PNPLA1–9. The sPLA2 family contains 10 catalytically active isoforms (IB, IIA, IIC, IID, IIE, IIF, III, V, X, XIIA) and 1 inactive isoform (XIIB) [54]. Table 1 presents the classification of the phospholipase A2 family.

**Table 1:** Classification of the PLA2 isozyme families present in human and mouse skin depending on cellular location and substrate specificity

Family	Isoforms	Substrate specificity	Location	References
cPLA2 calcium dependent	cPLA2-α	High specificity for PC, PE containing: arachidonic acid (cPLA2-IIα, cPLA2-Iiε, cPLA2-Iiζ) or linoleic acid (cPLA2-Iiδ, cPLA2-Iiε, cPLA2-Iiζ) at sn-2 position	Human immortalized keratinocytes (HaCaT)	[57]
	cPLA2-β		Murine keratinocytes (PAM212)	[58]
	cPLA2-γ			
	cPLA2-δ		Mouse keratinocytes	[59]
	cPLA2-ε		Human immortalized keratinocytes (HaCaT)	[60]
	cPLA2-ζ			
iPLA2 calcium independent	PNPLA1	High specificity for PE and PC at the sn-2 position (do not exhibit specificity toward any fatty acid)	Mouse keratinocytes Human fibroblasts	[61, 62]
	PNPLA2			
	PNPLA3			
	PNPLA4			
	PNPLA5			
	PNPLA6			
	PNPLA7			
	PNPLA8			
	PNPLA9		Normal human epidermal keratinocytes (NHEK)	[63]
sPLA2 calcium dependent	sPLA2-I *sPLA2-IB*	High affinity for anionic PL such as PE, PG, PS	Murine fibroblasts (NIH3T3)	[64]
	sPLA2-II *sPLA2-IIA* *sPLA2-IIC* *sPLA2-IID* *sPLA2-IIE* *sPLA2-IIF*	High affinity for anionic PL such as PE, PG, PS	Human immortalized keratinocytes (HaCaT) Human primary epidermal keratinocytes (HPEK) murine primary epidermal keratinocytes (MPEK)	[54]
	sPLA2-III	No data		
	sPLA2-V	High affinity for cell membrane **PC**		
	sPLA2-X	High affinity for cell membrane **PC**		
	sPLA2-XII *sPLA2-XIIA* *sPLA2-XIIB*	No data		

The cytosolic isoforms of PLA2 (cPLA2) primarily hydrolyze PC and PE, with cPLA2 isoenzymes exhibiting varied specificity in releasing fatty acids from the sn-2 position of phospholipids. The phospholipase cPLA2-IIα specifically releases AA, whereas other isoforms, including both cPLA2-IIβ and cPLA2-IIγ, lack fatty acid specificity. However, the cPLA2-IIδ isoform is a specific hydrolase for linoleic acid (LA), and the isoforms cPLA2-IIε and cPLA2-IIζ release both AA and LA from phospholipids [55, 56]. Isoenzymes from the iPLA2 family do not exhibit specificity toward any fatty acid [55].

Considering the metabolic changes in keratinocytes resulting from diverse phospholipase activities, it has been demonstrated that inhibition of cPLA2-α phospholipase limits the release of proinflammatory prostaglandin PGE2, thus alleviating inflammation and keratinocyte proliferation in human immortalized keratinocytes (HaCaT) [57]. Furthermore, it has been found that in HaCaT stimulated by proinflammatory cytokines (IL-17A, IL-17F, IL-1β, TNF-α, IL-6, IL-22), activation of the cPLA2ε isoform increases the generation of anti-inflammatory N-acylethanolamines (NAEs), which alleviate epidermal hyperplasia, skin swelling, and reduce the expression of psoriatic markers, such as S100A9 proteins [60]. Conversely, the opposite effect is observed during the deletion of the cPLA2ε gene [60], and inhibition of the cPLA2β isoform in murine keratinocytes (PAM212) suppresses cell proliferation and migration [58].

Isoenzymes from the sPLA2 family, such as sPLA2-IB and sPLA2-IIA, primarily hydrolyze anionic phospholipids, such as phosphatidylglycerols prostaglandin (PG), PS, and PE, but are practically inactive toward PC. Similar dependencies have been shown for sPLA2 isoforms II and III. However, sPLA2 isoforms V and X hydrolyze PC with significantly higher efficiency compared with other members of the sPLA2 family [65]. It has been demonstrated that both activation and inhibition of sPLA2 isoforms elicit biological effects in keratinocytes. Among sPLA2 phospholipases, the dominant isoform in keratinocytes, both human (human primary epidermal keratinocytes—HPEK) and mouse (murine primary epidermal keratinocytes—MPEK), is the sPLA2-IIF isoform. Its increased activity enhances the generation of plasmalogen-type lysoPE (P-LPE), which may lead to the development of epidermal hyperproliferative diseases, such as psoriasis or skin tumor induced by 9,10-dimethylbenz(a)anthracene/12-O-tetradecanoylphorbol-13-acetate (DMBA/TPA) [54, 66]. Additionally, it has been shown that the sPLA2-IB isoform of murine fibroblasts (NIH3T3) activates extracellular matrix metalloproteinase 2 (MMP-2) [64]. Moreover, it is known that the sPLA2 phospholipase isoform (sPLA2-IIA), by degrading bacterial cell membranes, prevents bacterial infections, and its genetic deletion leads, among other things, to the exacerbation of psoriasis in the distal areas of the mouse skin and weakening of carcinogenesis by reducing the activation of mast cells and increasing lymphocytes Th17 (Th17) immunity and macrophage infiltration [67]. Meanwhile, the isoform sPLA2 (sPLA2-IIE) localized in murine hair follicles regulates hair cell homeostasis [68].

Moreover, it has been shown that the phospholipase PNPLA1 belonging to the iPLA2 family in murine keratinocytes plays a crucial role in the biosynthesis of ω-O-acylceramide, a lipid component essential for the functioning of the skin barrier [61], and mutations in the PNPLA1 enzyme in both C- and N-terminal domains are associated with the development of autosomal recessive congenital ichthyosis (ARCI) [69, 70] causing abnormal lipid accumulation in fibroblasts and impairing the biosynthesis of ω-O-acylceramide [62]. However, other iPLA2 isoforms have not been thoroughly investigated regarding their metabolic actions at the level of skin [71].

### PLC

Another class of phospholipases catalyzing the hydrolysis of the ester bond between glycerol and the phosphate residue are the phospholipases PLC, which are activated by calcium ions, heterotrimeric large G proteins, small G proteins, and receptor/nonreceptor tyrosine kinases [72]. PLC are enzymes involved in the hydrolysis of PIP2, a minor phospholipid present in the cell membrane [73], which plays a crucial role in regulating many cellular processes. Reducing PIP2 levels is highly metabolically relevant because PIP2 acts as an activator of phospholipase D and phospholipase A2 and modulates actin polymerization by interacting with various actin-binding proteins and serves as a membrane-binding site for proteins containing pleckstrin homology (PH) domains [73, 74]. In addition to enzymatic activity, some PLC subtypes also function in other metabolic aspects, including: as a guanine nucleotide exchange factor, GTPase-activating protein, and adapter protein, independently of their lipase activity, thereby regulating cell polarization, cell cycle progression, and cell death [75]. Consequently the existence of 13 different PLC isoenzymes has been demonstrated, which are divided into six different groups, β, γ, δ, ε, ζ, and η, owing to the characteristic domains present in the isoenzymes. The common core of these isoenzymes includes a PH domain, a series of four EF-hands, a catalytic TIM barrel, and a C2 domain [72, 76] (Table 2).

**Table 2:** Classification of PLC isoenzyme families present in human and mouse skin depending on cellular location and substrate specificity

Family	Isoforms	Substrate Specificity	Location	References
PLCβ	PLCβ1 PLCβ2 PLCβ3 PLCβ4	Phosphoinositides (phosphatidylinositol and phosphatidylinositol phosphorylated derivatives)	Human keratinocytes, human fibroblast	[76–78]
PLCγ	PLCγ1 PLCγ2		Human keratinocytes	[76, 77]
PLCδ	PLCδ1 PLCδ3 PLCδ4		Human keratinocytes, human epidermal keratinocytes (NHEK)	[76, 77, 79]
PLCε			Primary mice cultured keratinocytes, epidermal mice keratinocytes and dermal mice fibroblasts	[76, 80, 81]

Individual PLC isoenzymes participate in various metabolic activities in skin. It has been found that inhibition of the PLCδ1 isoenzyme leads to a decrease in intracellular Ca^2+^ ion concentration and nuclear factor of activated T cells (NFAT) activity, as well as hyperactivation of mitogen-activated protein kinase (MAPK) protein kinase and inactivation of Ras-homolog of protein Ras homolog family member A (RhoA) in normal human epidermal keratinocytes (NHEK), which consequently may lead to a decrease in both ROS generation and inflammatory state [79]. Meanwhile, a crucial role in the development of skin inflammation is played by phospholipase PLCε, an isoenzyme that induces the production of proinflammatory cytokines, including IL-23, leading to the activation of IL-22-producing T cells (in transgenic murine keratinocytes K5-PLCe-TG) [80]. Increased expression of proinflammatory cytokines was also observed in the skin of mice with PLCδ1 knockout [82]. Furthermore, dysregulation at the phosphoinositide-specific phospholipase C (PI-PLC) level in the PI signaling transduction in human skin fibroblasts may lead to the development of melanoma [83].

### PLD

A class of phospholipases that hydrolyze the phosphodiester bond of phosphatidylcholine is phospholipase D, whose action leads to the formation of a signaling molecule, phosphatidic acid, which participates in many fundamental cellular processes such as vesicular transport, exocytosis, autophagy, and consequently regulates cellular metabolism, including contributing to tumorigenesis [52]. The two best-characterized mammalian isoforms of PLD are phospholipase D1 (PLD1) and phospholipase D2 (PLD2), which mainly differ in their N- and C-terminal regions in the polypeptide chain and their cellular localization. PLD1 is mainly localized in the Golgi apparatus, endosomes, and perinuclear region, while PLD2 is almost exclusively found in lipid raft fractions of the cell membrane [84]. Phospholipase D plays a dual role in cells as it is responsible for maintaining the integrity of cell membranes and participates in cell signaling, including the transport of intracellular proteins to the cell membrane surface [85, 86]. Moreover, phospholipase PLD1 is involved in the fibrogenesis of organs, such as the liver, heart, or lungs, as well as in autophagy [87]. On the other hand, PLD2 is involved in cell migration and proliferation owing to epidermal growth factor receptor (EGFR) synthesis, as well as in the formation and transport of secretory vesicles and endocytosis, and also in cytoskeleton rearrangement [85, 87, 88]. As a result, changes in the efficiency of phospholipase D activity affect cellular metabolism in various pathological conditions of the skin, and the application of 1α,25-dihydroxyvitamin D3 increases both gene expression and protein level of PLD1 in HaCaT keratinocytes [89]. It has been shown, among others, that the expression of both PLD1 and PLD2 is significantly higher in the epidermis and dermis of patients with psoriasis compared with healthy individuals [86]. Additionally, it has been found that in a mouse model of psoriatic skin inflammation induced by IL-23, genetic deletion of PLD2 has anti-inflammatory effects by reducing macrophage infiltration and decreasing the generation of two representative inflammatory markers, IL-6 and CXCL10 [86]. Increased activity of the glycerol transporter, keratinocytes aquaporin-3 (AQP3), located in membrane microdomains, leads to increased glycerol transport to PLD2, resulting in the differentiation of keratinocytes derived from ICR CD-1 outbred mice, as evidenced by increased expression of epidermal differentiation markers, such as keratin 1, keratin 10, and loricrin [90]. Activation of the glycerol channel AQP3 and increased activity of the PLD2 isoform were observed in murine epidermal keratinocytes isolated from wounded skin, which increased the generation of phosphatidylglycerol, consequently promoting cell membrane repair and wound healing [88, 91].

The highest activity of phospholipases has been demonstrated in keratinocytes; however, through the hydrolysis of phospholipids, phospholipases regulate inflammation and carcinogenesis processes in various skin cells, which can contribute to the development of skin pathological conditions when their activity is dysregulated [42, 92]. Table 3 presents the influence of changes in phospholipase activity on the metabolic response of skin cells.

**Table 3:** The impact of changes in phospholipases activity on the metabolic response of skin cells

Skin cells	Phospholipases activation/inhibition	Activator/inhibitor	Biological effect	Refs.
keratinocytes				
Human primary epidermal keratinocytes (HPEK) murine primary epidermal keratinocytes (MPEK)	sPLA2-IIF activation	1 mM Ca^2+^	P-LPE ↑ hyperproliferation ↓	[54]
Normal human epidermal keratinocytes (NHEK)	PLC-δ1 inhibition	Gene silencing	ROS and inflammation ↓ Ca^2+^ ↓; NFAT ↓ p38 MAPK ↑; RhoA ↓	[79]
Human immortalized keratinocytes (HaCaT)	cPLA2α inhibition	1-octadeca-2,6,9,12,15-pentaenylsulfanyl-propan-2-one (AVX001)	Reduction: - proinflammatory PGE2 level and inflammation - cell proliferation	[57]
Human immortalized keratinocytes (HaCaT)	cPLA2ε activation	Cytokines (IL-17A, IL-17F, IL-1β, TNF-α, IL-6, IL-22)	Anti-inflammatory NAE↑	[60]
Murine epidermal keratinocytes	PLD2 activation	PLD2/aquaporin-3 (AQP3) signaling	PG ↑ wound healing	[88]
Keratinocytes from ICR CD-1 outbred mice	PLD2 activation	PLD2/aquaporin-3 (AQP3) signaling	Differentiation ↑ proliferation ↑	[91]
Primary mice cultured keratinocytes	PLCε activation	Overexpressing PLCε	IL-23 ↑ Il-22 ↑	[80]
Murine keratinocytes (PAM212)	cPLA2β inhibition	Transfected si-PLA2G4B gene	Proliferation ↓ migration ↓	[58]
Fibroblasts				
Murine fibroblasts (NIH3T3)	sPLA2-IB activation	–	MMP2 ↑	[64]
Hair follicles				
Murine hair follicles	sPLA2-IIE inhibition	sPLA2-IIE gene silencing	Regulates hair homeostasis	[68]

AQP3-aquaporin-3; AVX001-1-octadeca-2,6,9,12,15-pentaenylsulfanyl-propan-2-one, *cPLA2* cytosolic calcium dependent phospholipase A2: *HaCaT* human immortalized keratinocytes, *HPEK* human primary epidermal keratinocytes *IL* interleukin, *MMP-2* matrix metalloproteinase-2, *MPEK* murine primary epidermal keratinocytes, *NAE* N-acylethanolamine, *NFAT* nuclear factor of activated T-cells, *NHEK* normal human epidermal keratinocytes, *NIH3T3* murine fibroblasts, *p38 MAPK* p38 mitogen-activated protein kinases, *PAM212* murine keratinocytes, *PG* phosphatidylglycerol, *PGE*_*2*_ prostaglandin E2, *PLA2* phospholipase A2, *PLC* phospholipase C, *PLD* phospholipase D, *P-LPE* plasmalogen-type lysoPE, *RhoA* Ras homolog family member A, *ROS* reactive oxygen species, *sPLA2* secretory phospholipase A2, *TNF-α* tumor necrosis factor α

### PUFAs

**Fig. 2:**
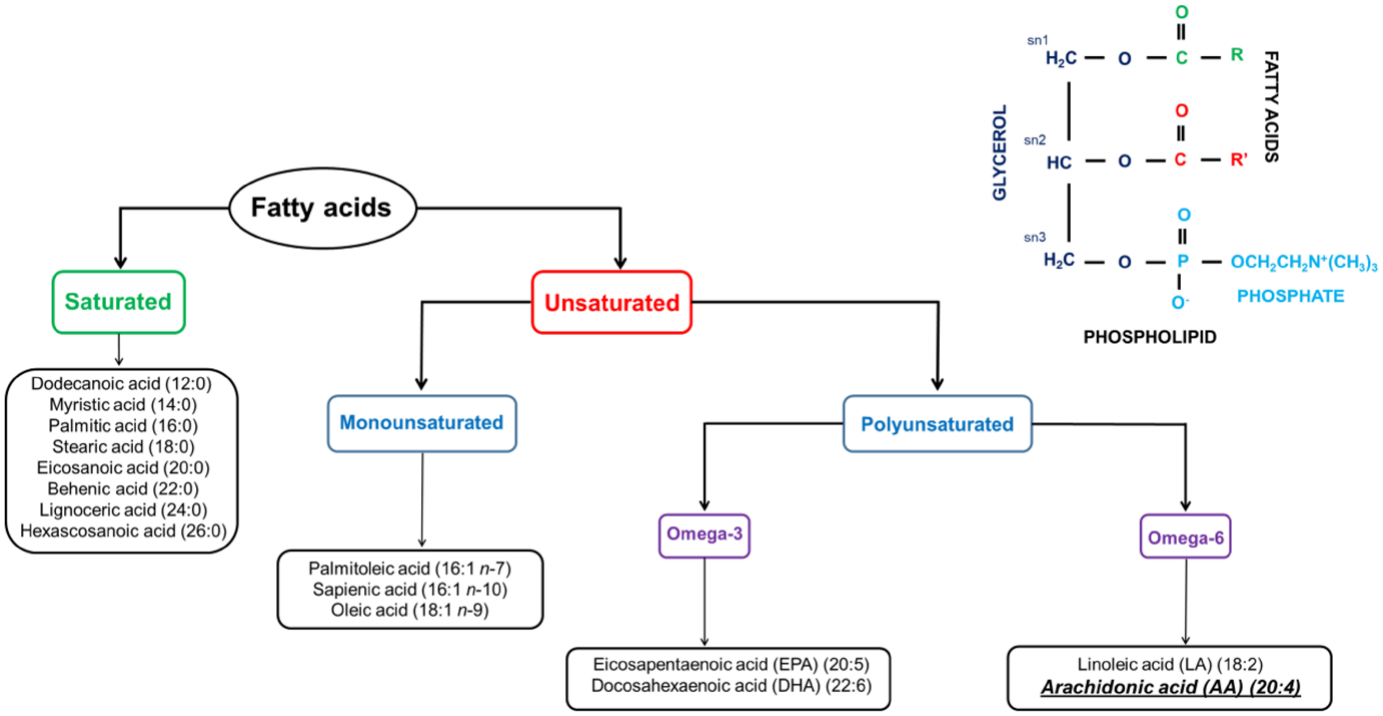
Fatty acids, both saturated and unsaturated present in human skin

Phospholipases, by hydrolyzing phospholipids, contribute to the release of PUFAs and intermediate products, such as endocannabinoids, which can then be metabolized mainly by FAAH and MAGL into free PUFAs [43]. Consequently, free fatty acids account for about 10–15% of the dry weight of healthy human epidermis [12]. Human skin contains many saturated and unsaturated fatty acids (Fig. 2), with arachidonic acid constituting approximately 6% of all free fatty acids [12]. However, literature data regarding the level of AA in primary epidermal cells—keratinocytes—are varied, from similar levels of AA and docosahexaenoic acid (DHA) in HaCaT cells [93] to significantly higher levels of AA compared with DHA and eicosapentaenoic acid (EPA) in human immortalized keratinocytes CDD 1102 KERTr (CRL2310) [53], which may be due to the origin of the cells. Free AA presence has also been demonstrated in human fibroblasts (CCD 1112Sk) and normal human dermal fibroblasts (NHDF); although its level is lower than other investigated PUFAs, such as DHA, EPA, and LA [94, 95].

However, since the quoted data concern skin cells obtained from cell culture, it should be remembered that the PUFA content in these cells also depends on the composition of the medium. The cited publications used media enriched with bovine serum [10% fetal bovine serum (FBS)], one of the components of which are PUFA (1% PUFA and 0.3% linoleic acid (18:2n-6) [96]. Moreover, since cells in culture easily take up lipids with the medium, it should be taken into account that this may have an impact on the level of cellular PUFAs, especially since the fatty acid level of cells in culture is approximately 2.5 times lower compared with cells in vivo, indicating the possibility of an impact PUFA derived from the medium on the level of fatty acids determined in cells from cell culture [96]*.*

### Free fatty acid metabolism

This review focuses only on the metabolism of arachidonic acid and bioactive metabolites, eicosanoids derived from arachidonic acid.

PUFAs undergo both nonenzymatic and enzymatic transformations. As a result of ROS-dependent peroxidation, PUFAs undergo oxidative fragmentation generating unsaturated aldehydes (4-HNE, MDA) [40]. Additionally, oxidative cyclization results in the formation of isoprostanes, isofurans, and isoketals [97].

**Fig. 3:**
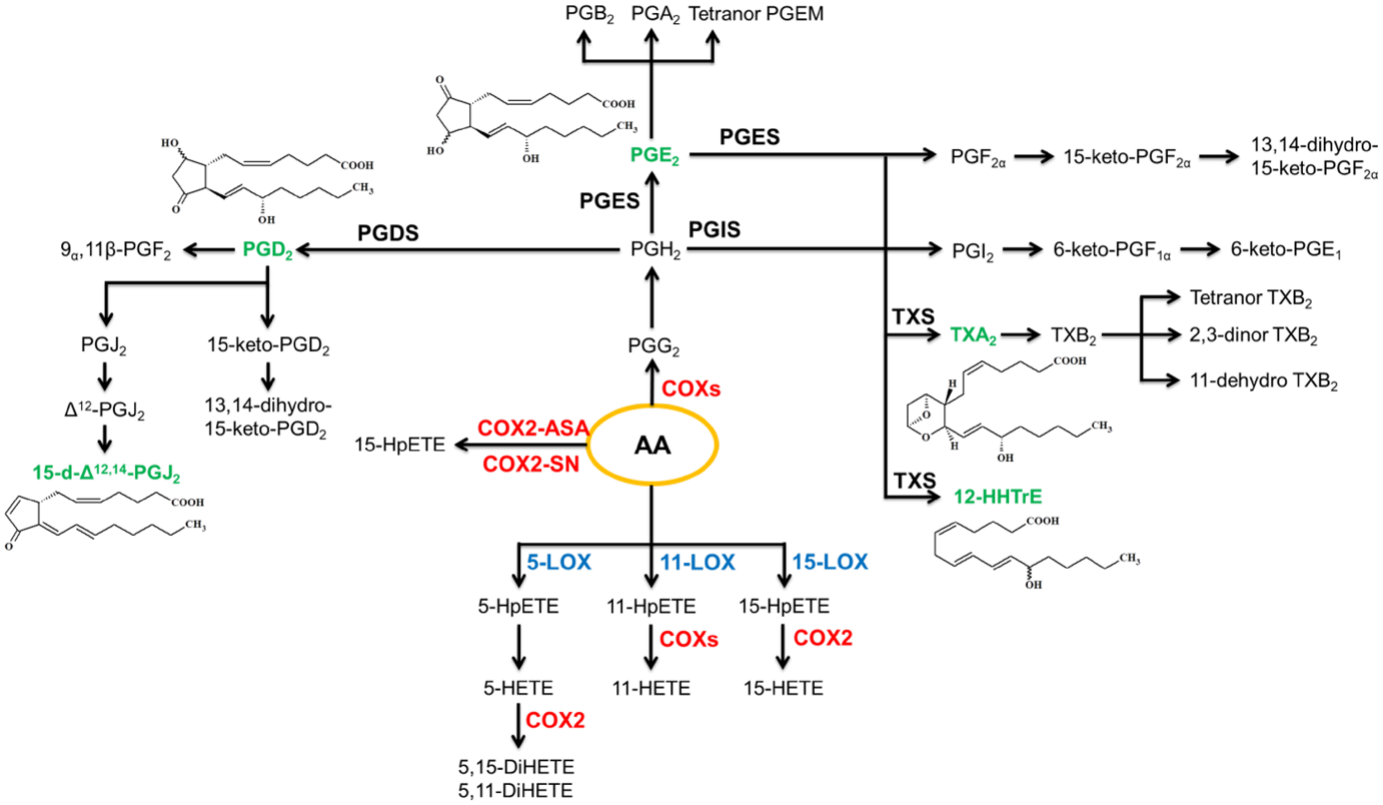
Metabolism of arachidonic acid under the action of cyclooxygenases (COXs). The eicosanoids marked in green have been discussed in detail in the later chapters. *12-HHTrE* 12-hydroxyheptadecatrienoic acid, *COX2-ASA* aspirin-acetylated COX-2, *COX2-SN* S-nitrosylated COX-2, *DiHETE* dihydroxy-eicosatetraenoic acid, *HETE* hydroxy-eicosatetraenoic acid, *HpETE* hydroperoxy-eicosatetraenoic acid, *LOX* lipoxygenase, *PGX* prostaglandin, *PGXS* prostaglandin synthase, *TX* thromboxane, *TXS* thromboxane synthase.

**Fig. 4:**
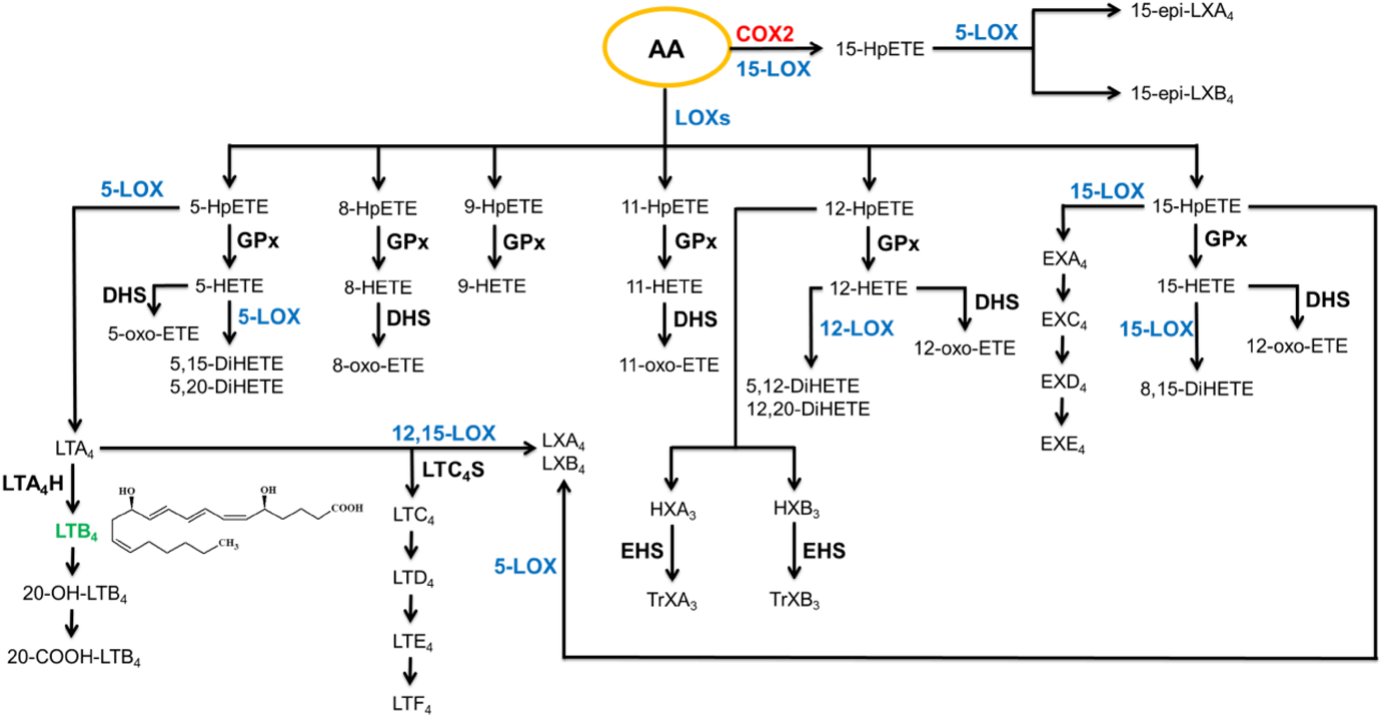
The metabolism of arachidonic acid under the influence of lipoxygenases (LOXs). The eicosanoids marked in green have been discussed in detail in the later chapters. *COX-2* cyclooxygenase 2, *DHs* hydroxyeicosanoid dehydrogenases, *DiHETE* dihydroxy-eicosatetraenoic acid, *EHS* epoxide hydrolases, *EX* eoxin, *GPx* glutathione peroxidase, *HETE* hydroxy-eicosatetraenoic acid, *HpETE* hydroperoxy-eicosatetraenoic acid, *HX* hepoxilin, *LT* leukotriene, *LTA*_*4*_*H* hydrolase LTA_4_, *LTC*_*4*_*S* synthase LTC_4_, *LX* lipoxin, *oxo-ETE* oxo-eicosatetraenoic acid, *TrX* trioxilin

**Fig. 5:**
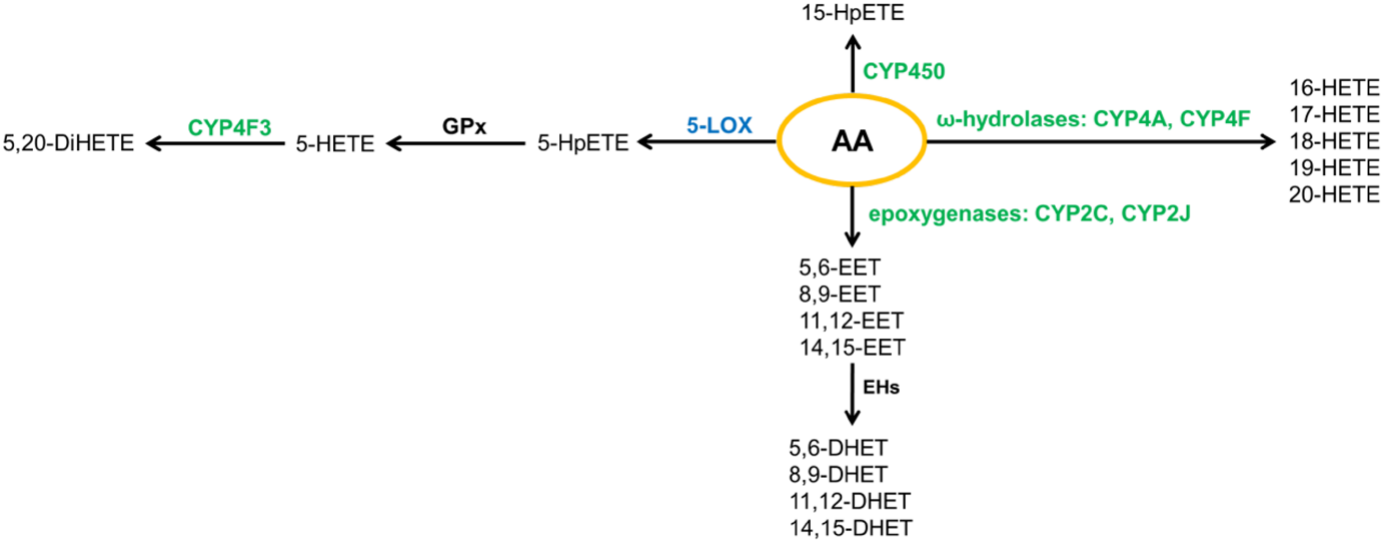
The metabolism of arachidonic acid involving cytochrome P450 (CYP450) isoenzymes. *DHET* dihydroxy-eicosatrienoic acid, *DiHETE* dihydroxy-eicosatetraenoic acid, *EET* epoxyeicosa-trienoic acid, *HETE* hydroxy-eicosatetraenoic acid, *HpETE* hydroperoxy-eicosatetraenoic acid, *LOX* lipoxygenase

The enzymatic metabolism of free PUFAs, such as AA, LA, DHA, or EPA, which occurs under the action of cyclooxygenases (COX1/2), lipoxygenases (LOXs), or cytochrome P450 (CYPs P450), leads to the formation of oxylipins, which are bioactive lipid mediators [98]. The type of oxylipin formed depends on the type of enzyme acting and the type of fatty acid being metabolized. The most frequently analyzed oxylipins, especially in skin, are derivatives of AA, known as eicosanoids [99]. In skin diseases, such as psoriasis and atopic dermatitis, there are changes in the levels of eicosanoids in response to metabolic changes resulting from the inflammatory conditions accompanying these skin diseases [32, 45, 98, 100] as well as environmental factors (e.g., UV radiation) [93]. The eicosanoid biosynthesis pathways are shown in Figs. 3, 4 and 5.

### COXs

In skin, as in other tissues of the human body, there are two isoforms of cyclooxygenases: the constitutive isoform, COX-1, which is constitutively expressed in most human tissues, including skin [101, 102] and the inducible isoform, COX-2 [103], which shares 60% amino acid homology with COX-1 [104]. COX-2 is induced by various stimuli, including exposure to proinflammatory factors such as growth factors, cytokines, carcinogens, endotoxins, and UV radiation [105, 106]. The highest levels of COX-2 are found mainly in suprabasal keratinocytes [103, 107].

Under the action of COXs, free arachidonic acid is metabolized to prostaglandin G2 (PGG_2_), which is an unstable product. The hydroperoxide group of PGG_2_ is reduced to an alcohol group by hemoperoxidase, generating prostaglandin H2 (PGH_2_). Then, through the action of prostaglandin synthase (PGXS) and thromboxane synthase (TXS), PGH_2_ is converted into a subclass of eicosanoids called prostanoids, which includes prostaglandins (PGD_2_, PGE_2_, PGI_2_) and thromboxane A2 (TXA_2_), all containing the prostanoid acid as a structural element. The activity of these enzymes also leads to the formation of 12-hydroxyheptadecatrienoic acid (12-HHTrE), which does not contain the prostanoic acid structure [108].

Prostanoids are rapidly metabolized, producing numerous metabolites, including the prostaglandin PGI_2_, which is formed by the action of PGI synthase on PGH_2_ and rapidly hydrolyzes to a stable but biologically inactive metabolite, 6-ketoprostaglandin F1α (6-keto PGF_1α_) [109]. As a result of dehydration of the hydroxyl groups of prostaglandins PGE_2_ and PGD_2_, the family of cyclopentenone prostaglandins (cyPGs) is formed. PGE_2_ undergoes dehydration to generate prostaglandin A2 (PGA_2_) and isomerization, resulting in prostaglandin B2 (PGB_2_) and 8-[(1R,2R,5R)−2-(2-carboxyethyl)−5-hydroxy-3-oxocyclopentyl]−6-oxooctanoic acid (tetranor PGEM). Similarly, during the dehydration of PGD_2_, prostaglandin J2 (PGJ_2_) is formed, which isomerizes into Δ12-PGJ_2_. The hydroxyl group (C-15) of Δ12-PGJ_2_ dehydrates to form 15-deoxy-Δ12,14-PGJ_2_ (15-d-PGJ_2_). Additionally, PGD_2_ is metabolized by dehydrogenase to 15-keto-PGD_2_, which is further metabolized by 15-keto-PG-Δ13-reductase to 13,14-dihydro-15-keto-PGD_2_. Moreover, PGD_2_ is metabolized by 11-ketoreductase to 9α,11β-PGF_2_ [109].

Regardless of the above metabolic transformations of prostaglandins, it has been observed that through the action of TXS on PGH_2_, active TXA_2_ is formed, which possesses an unstable ether bond that undergoes hydrolysis, generating the biologically inert thromboxane B2 (TXB_2_). TXB_2_, in turn, undergoes multifaceted metabolism, resulting in the β-oxidation pathway producing 2,3-dinor-TXB_2_, and the action of 11-hydroxythromboxane dehydrogenase generates 11-dehydro TXB_2_, which through its isomerization leads to the formation of tetranor TXB_2_ [109, 110]. Conversely, the action of enzymes blocking prostaglandin biosynthesis, such as aspirin-acetylated COX-2 and S-nitrosylated COX-2, leads to the generation of 15-hydroperoxy-eicosatetraenoic acid (15-HpETE) [111]. Oxidation of HpETE acids by COX-1 or COX-2 produces 11-hydroxy-eicosatetraenoic acid (11-HETE), as well as COX-2 action yields 15-HETE [108].

The enzymatic metabolism of arachidonic acid, including primarily COX-2-dependent prostaglandin synthesis, influences keratinocyte differentiation, hair follicle development, and hair growth [112]. Additionally, it is known that COX-2 mediates the development of inflammatory processes in the skin, while administering specific COX-2 inhibitors reduces the levels of inflammatory markers, such as macrophage inflammatory protein 2 (MIP-2) and TNF-α in skin with inflammatory hypersensitivity and nociception, as well as decreasing edema and vascular permeability [112]. Therefore, preparations containing COX-2 inhibitors are used topically in the treatment of skin diseases mediated by COX-2 [113].

### LOXs

Regardless of the metabolic activity of cyclooxygenases, mammalian skin possess a diverse set of non-heme iron-containing enzymes, such as LOXs, which also oxidize polyunsaturated fatty acids, including arachidonic acid. LOXs are classified on the basis of positional specificity in the oxidation of arachidonic acid (e.g., 5-, 8-, 12-, and 15-LOX); the tissue of their occurrence, such as platelet-type 12-LOX (p12-LOX), leukocyte-type 12-LOX (l12-LOX), and epidermal-type 12-LOX (e12-LOX). On the basis of the phylogenetic relationship of mammalian LOXs, they are divided into four subfamilies: 5-LOX, 12-LOX, and 12/15-LOX (reticulocyte-type 15-LOX-1 and leukocyte-type 12-LOX). Epidermal-type LOXs [12R-LOX, 15-LOX-2, 8-LOX, epidermis-type lipoxygenase-3 (LOX-3)] (Table 4) [114].

**Table 4:** Classification of the LOX skin human and mouse isoforms

Isoforms	Location	References
5-LOX	Human fibroblasts Human keratinocytes Human keratinocytes (HaCaT) Human Langerhans cells	[115–117]
8-LOX	Mouse keratinocytes	[118]
Platelet-type 12-LOX (p12-LOX)	Human keratinocytes	[119, 120]
Reticulocyte-type 15-LOX-1 15-LOX-2	Human keratinocytes (HaCaT), mouse keratinocyte	[118, 121]
LOX-3 (eLOX-3)	Mouse keratinocytes Human keratinocytes	[122, 123]

As a result of the action of lipoxygenases (LOXs: 5-, 8-, 9-, 11-, 12-, and 15-LOX) on arachidonic acid, the generation of hydroperoxyeicosatetraenoic acids (HpETEs; 5, 8, 9, 11, 12, and 15-HpETE) occurs, which are then transformed into hydroxyeicosatetraenoic acids (HETEs; 5, 8, 9, 11, 12, and 15-HETE) by glutathione peroxidase (GPx) (Fig. 4) [108, 124], which then undergo metabolism to oxo-eicosatetraenoic acids (oxo-ETEs) under the influence of hydroxyeicosanoid dehydrogenases (DHS), while under the action of LOXs they are transformed into dihydroxyeicosatetraenoic acid (DiHETE) [108]. Additionally, 5-LOX oxidizes 5-HpETE to leukotriene A4 (LTA_4_), which is metabolized to leukotriene B4 (LTB_4_) by LTA_4_ hydrolase (LTA_4_H), and C-20 LTB_4_ is subsequently hydroxylated to generate 20-hydroxy-leukotriene B4 (20-OH-LTB_4_), which is oxidized to 20-carboxy-LTB_4_ (20-COOH-LTB_4_) [125]. Conversely, LTC_4_ synthase (LTC_4_S) conjugates leukotriene A4 (LTA_4_) with glutathione (GSH) to form leukotriene C4 (LTC_4_), which serves as a precursor for leukotriene D4 (LTD_4_) and leukotriene E4 (LTE_4_) through sequential removal of amino acids from the glutathione residue [126]. Oxidation of LTA_4_ can also occur with the involvement of 12-LOX and 15-LOX, resulting in lipoxins, such as LXA_4_ and LXB_4_ [127]. 12-HpETE undergoes isomerization to hepoxilins (HXX_3_) through rearrangement of the-OOH group. So far, two hepoxilins have been identified, HXA_3_ and HXB_3_, both containing an epoxide group as well as an additional hydroxyl group. The epoxide group of hepoxilins is labile and undergoes hydrolysis under the action of epoxide hydrolases (EHS) or in acidic environments, forming trioxilin A3 or B3 (TrXA_3_, TrXB_3_), respectively [128].

On the other hand, the action of 15-LOX leads to the metabolism of 15-HpETE to eoxin A4 (EXA_4_) [108], which, analogous to the biosynthesis of leukotrienes, is conjugated with glutathione, forming eoxins C4, D4, and E4 (EXC_4_-EXE_4_) [129]. Additionally, under the influence of 5-LOX on 15-HpETE, lipoxins LXA_4_ and LXB_4_ are also formed, which are the first discovered lipid mediators exhibiting anti-inflammatory effects [127].

Considering the effects of AA metabolism involving LOXs, this group of enzymes is believed to play a significant role in modulating the proliferation and/or differentiation of epithelial cells, as well as in shaping inflammatory states, including inflammatory skin diseases and cancers, and wound healing [114]. Moreover, lipoxygenases also participate in maintaining the permeability barrier of the skin [130]. Mutations in LOXs genes in human epidermis have also been identified as the second most common cause of autosomal recessive congenital ichthyosis, while disruptions at the level of LOXs genes in mice led to the death of newborns owing to severely impaired skin barrier function [114, 130].

### CYP450

Another family of enzymes involved in the metabolism of AA to eicosanoids, but also responsible for the metabolism of sterols, steroids, vitamin A, and vitamin D, are the enzymes from the cytochrome P450 (CYP450) family [131], which are also the most versatile class of enzymes metabolizing xenobiotics, including drugs, both in the liver and extrahepatically, including in the skin [131, 132]. In the human body, 57 functional genes and 58 pseudogenes of CYPs from 18 different families and 44 subfamilies have been identified [133, 134]. CYPs have broad substrate specificity and catalyze various reactions including hydroxylation, heteroatom oxygenation, dealkylation, epoxidation, desaturation, and heme destruction among several others [135]. The classification of human cytochrome P450 enzymes is based on their functions or on the main class of substrates. Enzymes CYP1-4 are responsible for the biotransformation of xenobiotics, chemicals, and drugs, while enzymes from the CYP5 and CYP8 families participate in the biosynthesis of thromboxane and prostacyclin, and enzymes from the CYP11, CYP17, CYP19, and CYP21 families are involved in hydroxylation necessary for bile acid biosynthesis, metabolism of vitamin D3 and cholesterol, whereas CYP26 participates in the hydroxylation of retinoic acid [134]. For isoforms 2A7, 2S1, 2W1, 4A22, and 20A1, there is no definitive data yet [131]. Table 5 presents the family of isoenzymes present in human skin [136, 137].

**Table 5:** Classification of the cytochrome P450 isoenzymes family found in human skin and its cells based on main classes of compounds metabolized by CYPs

Isoforms	Classes of compounds metabolized by CYPs	References
1B1, 7B1, 8B1, 11A1, 11B1, 17A1, 19A1, 21A2, 27A1, 39A1, 46A1, 51A1	Sterols	[131, 136, 137]
1A1, 1A2, 2A6, 2A13, 2B6, 2C8, 2C9, 2C18, 2C19, 2D6, 2E1, 2F1, 3A4, 3A5, 3A7	Xenobiotics	
2J2, 2U1, 4B1, 4F11, 4F12, 4F22, 4V2, 4X1	Fatty acids	
4F2, 4F3, 4F8	Eicosanoids	
2R1, 24A1, 26A1, 26B1, 26C1	Vitamins	
2A7, 2S1, 2W1, 4A22, 20A1	Unknown	

Isoforms	Location	References
1A1, 1B1, 2B6, 2E1, 3A5	Human keratinocytes	[140]
4F22	Human keratinocytes	[141]
11A1	Human keratinocytes	[142]
1A1, 1B1, 2E1, 3A5, 3A7	Human Langerhans cells	[143]
1A1, 1B1, 2A6, 2D6, 2E1, 3A5, 3A7	Human fibroblasts	[143]
1A1, 1B1, 2A6, 2E1	Human melanocytes	[143]

Cytochrome P450 isoenzymes also participate in the generation of eicosanoids from AA (Fig. 5). Nevertheless, metabolites resulting from the action of CYP450 are much less studied than metabolites from other enzymatic pathways. Under the action of ω-hydroxylases (CYP4A, CYP4F), AA undergoes metabolism to HETEs, while the action of CYP2C and CYP2J epoxygenases leads to the formation of epoxyeicosatrienoic acids (EETs). EETs are further metabolized by epoxide hydrolases (EHS) to dihydroxyeicosatrienoic acids (DHETs) [44, 138]. However, it has also been demonstrated that the action of CYP450 enzymes on arachidonic acid leads to the formation of 15-HpETE, and ω-oxidation of 5-HETE, a metabolite resulting from the action of 5-LOX and GPx, by CYP4F3 results in the formation of 5,20-diHETE [111, 139].

In human keratinocytes, expression of constitutive isoforms of CYP1A1, CYP1B1, CYP2B6, CYP2E1, and CYP3A5 has been detected [140]. It has also been observed that after induction by the glucocorticosteroid—dexamethasone, which has strong anti-inflammatory, antiallergic, and immunosuppressive effects and is used in dermatology for conditions, such as psoriasis and allergic skin diseases, the expression of CYP1A, CYP2B, CYP2E, and CYP3A is increased at the mRNA level [140, 144]. Additionally, psoriasis and UVA radiation promote a significant increase in the expression of the CYP2S1 isoform in psoriatic skin lesions compared to healthy skin [145]. Moreover, increased enzymatic activity of CYP1A1 has been demonstrated in the skin of psoriasis patients, which affects the reduction of aryl hydrocarbon receptor (AHR) pathway activation, which acts anti-inflammatory [146]. Owing to the inhibition of cytochrome P4503A4 metabolism, dexamethasone, a corticosteroid inhibiting metabolism in the epidermis, may be used in the topical treatment of skin diseases such as skin allergies, atopic dermatitis, and psoriasis [147]. Another isoform, CYP4F22, is a ω-hydroxylase responsible for ceramide synthesis and participates in mechanisms of skin permeability barrier formation [141].

### PUFA metabolites and their functions in the skin

The metabolites of free fatty acids are oxylipins, a subgroup of which are eicosanoids, which are derivatives of arachidonic acid and produced by all types of skin cells [45, 148]. These compounds are not stored in cells but are synthesized and released in response to chemical and physical stimuli as well as various skin disease states. Oxylipins play an integral role in the mechanisms of structural and metabolic homeostasis of skin. However, the most frequently analyzed metabolites are eicosanoids owing to the widespread occurrence of AA and consequently higher concentrations of eicosanoids in the human body. Eicosanoids reveal mainly pro-inflammatory effects, while metabolites of other fatty acids, including EPA and DHA, have mainly anti-inflammatory effects [149, 150]. However eicosanoids mediate the regulation of inflammation caused mainly by environmental factors such as UV exposure, but also inflammatory or allergic disease pathologies, including psoriasis and atopic dermatitis [45, 100]. Changes in eicosanoid levels depend on the type of disease, its severity, as well as the individual characteristics of the patient and the location of these compounds in the skin.

Table 6 presents the directions of changes in eicosanoid levels influenced by physiological processes, diseases, and therapies, observed both in the skin and in epidermal and dermal cells, including those from healthy individuals and patients with skin diseases.

**Table 6:** Changes in the level of eicosanoids as a result of physiological processes, diseases, or therapies

Factors inducing metabolic changes	Tissue/Cells	Eicosanoids	References
Skin aging	Human skin	PGE_2_↑	[151]
DHA (50 µM) for 3 days	Human dermis, human epidermis	PGE_2_↓ 12-HETE↑	[148]
Sodium lauryl sulfate (24 h postpatch removal)	Human epidermis	TXB_2_↑ 12-HETE↑	[152]
IL-1β (10 ng/mL) for 24 h	Normal human dermal fibroblasts	PGE_2_↑, PGB_2_↑, PGA_2_↑, TXB_2_↑ 9-HETE↑, 11-HETE↑	[95]
Atopic dermatitis	Human skin	PGE_2_↑, PGJ_2_↑, PGF_2α_↑, LTB_4_↑, 5-HETE↑, 8-HETE↑, 11-HETE↑	[32]
Psoriasis	Human skin	5-HETE↑, 12-HETE↑, 15-HETE↑, 5S,15S-diHETE↑	[153]
Psoriasis	Human dermis	PGE_2_↑, 15-HETE↑	[154]
Psoriasis	Mononuclear cells of patients with psoriasis	15-d-PGJ_2_↓, TXB_2_↑, LTB_4_↑, 15-HETE↓,	[155]
Androgenetic alopecia	Bald scalp of men	PGE_2_↓, PGD_2_↑, 15-d-PGJ_2_↑	[156]

The biological effects of eicosanoids depend on both their cellular localization and the type of membrane receptor they activate, as the metabolic activity of eicosanoids primarily occurs through the activation of specific G protein-coupled membrane receptors [45, 157]. These receptors represent the largest and highly diverse group of membrane proteins responsible for transmitting signals across the lipid bilayer to effector sites within the cell [157]. Some receptors, such as the prostaglandin F receptor (FP), prostaglandin I2 receptor (IP), and thromboxane receptor (TP), are activated by only one lipid ligand, while others, including prostaglandin E2 receptors (EP1, EP2, EP3, EP4), prostaglandin D2 receptors (DP1, DP2), and cysteinyl leukotriene receptors (CysLT1, CysLT2), can bind to various ligands, thereby having multifaceted effects on cellular metabolism [45, 157]. Additionally, eicosanoids can also act as ligands for receptors such as peroxisome proliferator-activated receptors (PPARs), transient receptor potential cation channel subfamily V member 1 (TRPV1) and transient receptor potential cation channel, subfamily A, member 1 (TRPA1), which are activated by other compounds as well [157, 158].

## Prostaglandins

### PGE_2_

PGE_2_, generated in a COX-dependent pathway (Fig. 3) both in epidermal keratinocytes and dermal fibroblasts, exhibits strong proinflammatory and vasodilatory properties. Additionally, it promotes proliferation and modulates the immunosuppression of cells [159, 160]. PGE_2_ also participates in the proliferation and differentiation of keratinocytes and, by activating EP1-EP4 receptors, directly influences epidermal barrier functions [161, 162].

As a result of EP1 receptor activation in adult primary human keratinocytes, PGE_2_ promotes the formation of corneocytes, dead cells that create a protective barrier preventing harmful substances/pathogens from penetrating the skin as well as protecting against injuries and UV radiation. Additionally, PGE_2_ is involved in the induction of differentiation proteins, such as cytokeratin K10 and transglutaminase, which are responsible for cell division and differentiation, transport, and cell adhesion. These processes are inhibited by two known EP1 receptor antagonists (SC51322 and AH6809) [163]. In normal human dermal fibroblasts (NHDFs), PGE_2_ also increases extracellular signal regulated kinase 1/2 (ERK1/2) phosphorylation and intracellular Ca^2+^ concentrations via EP1 receptor activation. This leads to a decrease in the type 1 collagen expression and an increase in the extracellular matrix metalloproteinase 1 (MMP-1) expression, resulting in accelerated skin aging [164].

In contrast, activation of EP2 receptors by PGE_2_ in strains of human dermal fibroblasts leads to increased regulation of cyclic adenosine monophosphate (cAMP), which contributes to the reduction of fibroblast contractility and consequently reduces skin scarring [165]. Additionally, it was found that increased PGE_2_ production resulting from topical application of a mycotoxin (alternariol-AOH) to mouse skin leads to activation of the EP2 receptor, which increases cAMP levels promoting phosphorylation of the cAMP response element-binding protein (CREB) transcription factor, which regulates the transcription of genes responsible for the proliferation of primary mouse keratinocytes (PMK) [166]. On the other hand, activation of the EP2 receptor by its agonist, such as butaprost, a structural analogue of PGE_2_, leads to the formation of the β-arrestin1-Src complex, which activates the EGFR and its effectors, including p-ERK1/2, phosphorylated signal transducer and activator of transcription 3 (p-STAT3) and phosphorylated protein kinase B (p-Akt), and also increases the activity of cyclic AMP-dependent protein kinase (PKA) and its downstream effectors, including phosphorylated glycogen synthase kinase 3 (p-GSK3), p-CREB and p-ERK1/2. The above signaling pathways enhance the proliferation of murine keratinocytes, which has been suggested to contribute to the induction of skin cancer [167].

It is also suggested that PGE_2_ participates in two mechanisms responsible for melanocyte dendrite formation. Activation of proteinase-activated receptor 2 (PAR-2) in human neonatal keratinocytes stimulates the release of PGE_2_, leading to the activation of EP1 and EP3 receptors in melanocytes and the activation of protein kinase C zeta (PKCζ), resulting in melanocyte dendrite formation [168, 169]. Additionally, it has been shown that activation of EP3 by a synthetic agonist of EP3 receptors (ONO-AE-248) suppresses the generation of chemokine (C-X-C motif) ligand 1 (CXCL1), which recruits neutrophils, induced by TNF-α administration in normal human epidermal keratinocytes. Similar dependencies have been demonstrated in a mouse model of contact hypersensitivity [170]. Therefore, it can be suggested that regulation of EP3 receptor signaling in keratinocytes may be a therapeutic target in the treatment of skin inflammation.

Moreover, it has been demonstrated that through EP2 and EP4 receptors, PGE_2_ can also play an anti-apoptotic role, as observed in mouse skin exposed to UVB radiation. Agonists of both receptors restore the activation of PKA and Akt, reducing apoptosis by approximately 50% in dorsal skin mice [171]. Activation of EP2 and EP4 receptors by PGE_2_ induces PKA signaling and AHR receptor activation in T lymphocytes (Th17 and Th22) of C57BL/6 mice, promoting the release of IL-22, which may lead to allergic contact dermatitis (ACD) [172]. PGE_2_ also affects the interaction between skin layers. IL-1 generated by keratinocytes increases PGE_2_ levels in fibroblasts, leading to enhanced keratinocyte proliferation [173]. Furthermore, a complex feedback loop was observed in co-culture of fibroblasts and dendritic cells (DCs). This mechanism involves the secretion of IL-1 and TNF-α in DCs, promoting increased PGE_2_ generation in fibroblasts, and subsequently leading to increased release of IL-23 in DCs, resulting in Th17 cell expansion [174]. On the other hand, other studies have shown that activation of EP2 and EP4 receptors by PGE_2_ in cultured naïve CD4 lymphocytes T cells (CD4 + CD25) inhibits transforming growth factor beta (TGF-β) signaling, which is responsible for the differentiation of iTreg cells necessary for controlling inflammatory processes through the generation of proinflammatory IL-22 [175].

Furthermore, it has been found that activation of EP2 receptors in murine keratinocytes (PAM212) leads to inhibition of PAR2 receptor activity, which consequently inhibits thymic stromal lymphopoietin (TSLP) expression. Therefore, it is suggested that this mechanism may represent a novel therapeutic strategy in the treatment of atopic dermatitis (AD) [176]. On the other hand, the PGE_2_ metabolite, 13,14-dihydro-15-keto-PGE_2_, through activation of EP4 receptors, induces Axl phosphorylation via receptor tyrosine kinase (RTK), leading to activator protein 1 (AP-1) transactivation, which increases oncostatin M (OSM) generation in human macrophages. OSM is a cytokine with strong anti-inflammatory properties, reducing TNF-α and IL-1β expression and thereby accelerating wound healing [177].

Changes in PGE_2_ levels have also been analyzed in the context of psoriatic skin. However, data obtained indicate both a lack of changes in PGE_2_ levels in psoriatic skin compared with healthy skin [153], and a significant increase in the level of PGE_2_ in both psoriatic skin and blood mononuclear cells of patients with psoriasis [155, 178]. Consequently, attempts were also made to treat persistent vitiligo through intradermal administration of PGE_2_ or PGF_2α_ supported by NB-UVB phototherapy. In the case of using PGE_2_, it resulted in a significantly earlier onset of repigmentation and a higher degree of healing in the treated areas compared with the use of PGF_2α_ [179].

### PGD_2_ and 15-d-PGJ_2_

Prostaglandins, which are potent mediators of inflammatory and immune responses in human skin, are also important effector molecules in cellular responses to cytokines. However, the direction of action of prostaglandins depends on various factors, including the affected organ/tissue or the receptor they activate and the specific physio/pathological situation. Langerhans cells and mast cells are considered to be the main sites of production of prostaglandins PGD_2_ and 15-d-PGJ_2_ in the skin [159, 180]. Both prostaglandins are also generated in keratinocytes and fibroblasts [181–184]. It was found that as a result of dehydration, PGD_2_ is converted into prostaglandin J2, which isomerizes to Δ12-PGJ_2_, and its hydroxyl group (C-15) is dehydrated with the generation of 15-deoxy-Δ12,14-PGJ2 (15-d-PGJ_2_) [109] (Fig. 3). The action of PGD_2_ and 15-d-PGJ_2_ is related to the activation of DP1 and DP2 receptors, respectively [185].

PGD_2_ is involved in immune and allergic reactions, has strong antiproliferative properties, and regulates inflammation [182–184]. It has been shown that increased activity of PLA2 type III (PLA2G3) is characteristic of mast cells cultured with fibroblasts, which promotes an increase in the activity of PGD_2_ synthase in fibroblasts, which stimulates the maturation of mast cells by activating DP1 receptors [186]. Additionally, PGD_2_-DP2 signaling, demonstrated after bee venom injection, promotes the production of IgE antibodies, causing dendritic cells to migrate to lymph nodes, contributing to the development of acquired immunity [187]. In contrast, topical application of PGD_2_ inhibits hair follicle neogenesis in mice and humans by activating DP2 receptors [156].

Furthermore, PGD_2_, through activation of AP-1 in human foreskin keratinocytes, increases the mRNA expression of beta-defensin 3 (hBD-3), a peptide with antimicrobial properties; this effect is suppressed by ramatroban, a DP2 receptor antagonist [182]. However, primary human keratinocytes (GM22251) exposed to PGD_2_ show an increased ability to convert the androgen androstenedione into testosterone, which may be a potential therapeutic target for patients with androgenetic alopecia (AGA) [188]. Additionally, PGD_2_-induced testosterone production is believed to be mediated by ROS, as evidenced by increased production of the unsaturated 4-hydroxynonenal aldehyde and decreased testosterone levels following administration of the antioxidant N-acetylcysteine (NAC) with PGD_2_ [188]. In human dermal papilla cells (hDPCs), PGD_2_, through DP2 receptors involved in the signaling pathway downstream of various inflammatory mediators, glycogen metabolism, cell proliferation, and apoptosis, also enhances androgen receptor (AR) signaling and AKT activation (Jeong et al., 2018), involved in the signaling pathway downstream of various inflammatory mediators, glycogen metabolism, cell proliferation, and apoptosis [189]. Additionally, it has also been recognized that the PGD_2_-DP2 interaction leads to increased production of chemokines from macrophages (MDCs) and lymphocytes (RANTES), which play an important role in the development of chronic allergic dermatitis, such as IgE-mediated very late-phase (vLPR) response and chronic hypersensitivity contact dermatitis (CHS) [184], which was confirmed in a study in which DP2 receptor inhibition reduced chemokine expression in recurrent IgE-induced dermatitis [190].

Increased levels of prostaglandin PGD_2_, produced by mast cells, was also observed in atopic dermatitis [191, 192]. Additionally, it was indicated that this prostaglandin may have an effect in two directions, namely activation of DP1 receptors, which reduces inflammation and preserves the barrier function of the skin [193], or activation of DP2 receptors, leading to the induction of chemotaxis in leukocytes and promoting the development of inflammation [194]. It was also shown that reducing PGD_2_ levels can inhibit allergic dermatitis in patients with atopic dermatitis [195]. However, a phase II clinical trial evaluating the effect of the DP2 receptor antagonist timapiprant in atopic dermatitis (NCT02002208) did not show any significant improvement [196] (Tables 7 and 8)

**Table 7:** Metabolic effects of PGE_2_ and activation/inhibition of its receptors on skin cells

Ligand	Receptor	Tissues/cells	Action/mechanism	References
Keratinocytes				
PGE_2_	Not examined	Human epidermal Keratinocytes	Proliferation and differentiation of keratinocytes	[162]
PGE_2_	EP2	Alternariol induced proliferation in primary mouse keratinocytes (PMK)	Activation of EP2/cAMP/p-CREB signaling cascade	[166]
PGE_2_	EP2	Mouse keratinocytes (PAM212) in murine atopic dermatitis	Inhibition of thymic stromal lymphopoietin (TSLP)	[176]
EP1 anatagonists SC51322 and AH6809	EP1	Primary human keratinocytes	Inhibition of the synthesis of corneocytes, cytokeratin K10 and epidermal transglutaminase	[163]
EP2 agonist butaprost	EP2	Mice epidermal keratinocytes	Activation of the EGFR and PKA pathways induces cell proliferation	[167]
EP3 agonist AE248	EP3	Normal human epidermal keratinocytes	CXCL1 ↓	[170]
Fibroblasts				
PGE_2_	EP1	Normal Human dermal fibroblasts (NHDFs)	Activation of p-ERK ½ Ca^2+^ ↑ COL1A1 ↓ MMP1↑	[164]
PGE_2_	EP2	Strains of human dermal fibroblasts	cAMP ↑	[165]
PGE_2_	Not examined	Treating newborn human foreskin fibroblasts of IL-1	Keratinocyte proliferation	[173]
PGE_2_	Not examined	Human fibroblasts	Increasing the release of IL-23 in dendritic cells, which causes Th17 expansion	[174]
***Melanocytes***				
PGE_2_	EP1, EP3	Cultured human melanocytes	Melanocyte dendrite formation	[168, 169]
Lymphocytes				
PGE_2_	EP2, EP4	Th17, Th22 allergic contact dermatitis (ACD)	IL-22 release	[172]
PGE_2_	EP2, EP4	Mouse CD4 + CD25—naive T cells	Inhibits T cell conversion by antagonizing TGF-β signaling	[175]
**Skin**				
PGE_2_	EP2 + EP4	Mouse dorsal skin exposed to UVB radiation	Anti-apoptotic effect due to activation of cyclic AMP-dependent protein kinase (PKA) and protein kinase B (Akt)	[171]

**Table 8:** Metabolic effects of PGD_2_ and activation/inhibition of its receptors on skin cells

Ligand	Receptor	Tissues/cells	Action/mechanism	References
Keratinocytes				
PGD_2_	Not examined	Human neonatal foreskin keratinocytes	Peptide hBD-3↑	[182]
PGD_2_	Not examined	Primary human keratinocytes (GM22251) androgenic alopecia (AGA)	Testosterone↑ 4-HNE↑	[188]
Fibroblasts				
PGD_2_	DP1 activation	Swiss 3T3 fibroblasts	MC cell maturation	[186]
**Other cells**				
PGD_2_	DP2 activation	Human dermal papilla cells (hDPCs)	Activates androgen receptor (AR) and AKT signaling	[199]
PGD_2_	DP2 activation	Human and mouse hair follicles	Inhibition of neogenesis	[156]
Skin				
PGD_2_	DP2 reduction	Earlobes	Reduces ear swelling and chemokine expression	[190]
PGD_2_	DP2 activation	Mouse skin	Production of BV-specific IgE antibodies causing migration of dendritic cells (DC)	[187]

PGD_2_, as well as its metabolite 15-d-PGJ_2_, when used in in vitro studies, inhibit the growth of human hair follicles explanted and hair growth in mice through the activation of the DP2 receptor [181]. Additionally, 15-d-PGJ_2_ induces apoptosis in follicular keratinocytes, as evidenced by increased expression of the pro-apoptotic Bcl-2-associated X protein (BAX) and caspase 3, as well as decreased expression of the anti-apoptotic protein B-cell lymphoma 2 (Bcl-2) [181]. This prostaglandin also arrests the cell cycle in the G1 phase of keratinocytes, thereby inhibiting cell proliferation [197]. In contrast topical application of 15d-PGJ_2_ on skin wounds in diabetic mice increases the expression of peroxisome proliferator-activated receptor gamma (PPARγ) in macrophages at the wound site, thus reducing inflammation and accelerating wound closure [198].

Prostaglandins are potent mediators of inflammatory and immune responses in human skin and are important effector molecules in cellular responses to cytokines. The action of prostaglandins depends on various factors, including the organ or tissue involved, the receptor to which they attach, and the bodily function or physiological situation [164, 166, 187].

### Thromboxane A2

The metabolite of arachidonic acid generated by the action of COX and TX synthase (Fig. 3) is thromboxane TXA_2_, which under physiological conditions TXA_2_ is synthesized in very small amounts, and most studies indicate that its level assessed by liquid chromatography with tandem mass spectrometry (LC–MS/MS) methods is below the detection limit [200]. However, during various pathologies, its concentration undergoes a rapid increase. TXA_2_ has an unstable ether linkage, which undergoes hydrolysis under aqueous conditions, generating the biologically inert TXB_2_. TXA_2_ primarily acts through the activation of the thromboxane A2 receptor (TP), which is found in various tissues and cells, including in the vascular system (smooth muscle cells and endothelium), blood platelets, lungs, kidneys, heart, thymus, and spleen [201].

It has been found that the generation of TXA_2_ significantly increases immediately after skin injury and participates in blood platelet aggregation [202, 203], which is necessary to stop bleeding and promote effective wound healing [204, 205]. In a murine model of skin inflammation, it has been demonstrated that TXA_2_ generated by blood platelets, through TP receptor activation, induces the synthesis of the proinflammatory cytokine interleukin 6 and PGE_2_, and also suppresses the expression of the anti-inflammatory marker, mannose receptor C type 1 (CD206) in macrophages [206]. TXA_2_ generated by mouse keratinocytes through TP receptor activation triggers skin itching in murine atopic dermatitis [207]. Additionally, it has been shown that intradermal injection of IL-31 in mice increases intracellular Ca^2+^ levels in keratinocytes, which also leads to an increase in TXA_2_ concentration. TXA_2_, by activating TP receptors, induces skin itching in mice [208]. Elevated levels of TXA_2_ have also been demonstrated in population studies of patients with atopic dermatitis, which may be responsible for the course of the disease [209]. In other studies, it has been shown that the TXA_2_-TP signaling pathway participates in the development of psoriatic skin inflammation in mice induced by imiquimod (IMQ). This mechanism involves increased generation of the proinflammatory IL-17A in Vγ4 + γδ T cells, promoting psoriatic skin inflammation in mice [210].

### Leukotrienes, including LTB_4_

Short-lived lipid mediators include leukotrienes (LTs) (Fig. 4), which act in an autocrine and paracrine manner, and their concentration increases in skin during inflammatory diseases [211]. Leukotrienes are divided into two groups: the first represented by LTB_4_ and the second comprising cysteinyl leukotrienes (cys-LTs), namely LTC_4_, LTD_4_ and LTE_4_ [212, 213]. Leukotrienes are not stored in cells but are rapidly synthesized de novo and released within minutes of cell activation. Various stimuli induce the generation of leukotrienes, including immunological and proinflammatory stimuli [213]. LTB_4_ acts through the activation of leukotriene B4 receptor 1 (BLT1), while leukotrienes C4, D4, and E4 primarily act through the activation of CysLT1 and CysLT2 receptors [214].

LTB_4_, a metabolite of arachidonic acid (Fig. 4), is a classic lipid mediator of inflammation primarily produced by blood cells, such as leukocytes, granulocytes, macrophages, and dendritic cells, in the epidermis and dermis, as well as during host defense against pathogens and in patients with allergies, autoimmune diseases, and metabolic disorders [215, 216]. LTB_4_ has high affinity for the BLT1 receptor and low affinity for the leukotriene B4 receptor 2 (BLT2) [217]. It has been found that during postoperative incisional pain, activation of BLT1 receptors by LTB_4_ exacerbates pain responses by promoting local infiltration of inflammatory monocytes and cytokine production (IL-6, IL-1β, and TNF-α) [218]. Furthermore, increased generation of LTB_4_ is responsible for leukocyte recruitment to sites of tissue damage and infection [215].

Bacterial skin infection caused by methicillin-resistant Staphylococcus aureus (MRSA) has been observed to induce macrophage activation, leading to increased LTB_4_ production and neutrophil recruitment in diabetic mice; therefore, treatment with a BLT1 receptor antagonist reduces neutrophil recruitment and lowers chemokine/cytokine levels [intercellular adhesion molecule 1 (ICAM1), chemokine (C-X-C motif) ligand 2 (CXCL2)] [219]. Moreover, during MRSA infection, prevention of the LTB_4_/BLT1 pathway action is a promising therapeutic strategy that inhibits inflammatory responses by inhibiting transcriptional pathways involved in enhancing inflammasome expression [220]. On the other hand, local application of LTB_4_ in difficult-to-treat skin infections intensifies both the function of local antibacterial effectors and inflammatory response [220].

It has been shown that the production of LTB_4_ increases significantly in the skin of patients with psoriasis, which is associated with increased neutrophil infiltration and T cell activity, leading to increased LTB_4_ synthesis in response to increased 5-LOX activity [221]. However, activation of BLT1 receptors by LTB_4_ leads to the activation of CXCL1, CXCL2, and C-X-C motif chemokine receptor 2 (CXCR2) receptors in neutrophils, accelerating their infiltration. In dendritic cells and lymphocytes, activation of BLT1 receptors facilitates migration and production of cytokines IL-17 and IL-23, contributing to the development of psoriasis [222, 223]. Additionally, LTB_4_ is also considered an important signaling molecule involved in skin pigmentation in human melanocytes [224]. It has also been shown that LTB_4_ has an antiproliferative effect on melanocytes in both healthy individuals and those with acquired vitiligo [225].

In the skin of patients with atopic dermatitis, increased production of LTB_4_ was observed, as well as higher activity of leukotriene-A4 hydrolase in the peripheral blood cells of these patients [226, 227]. However in skin lesions associated with atopic dermatitis or allergic contact dermatitis was indicated that LTB_4_ increases generation of the chemokine CCL27 induced by TNF-α in human keratinocytes [228]. A pilot study of oral therapy with a 5-LOX inhibitor (zileuton) for AD demonstrated that the compound prevented disease exacerbations and prolonged remission, confirming the functional role of LTB_4_ in the development of this disease [229]. Considering the significance of both proinflammatory leukotrienes and prostaglandins in the pathophysiology of asthma, inhibition of cPLA2 activity is believed to inhibit the generation of these eicosanoids [230]. Completed phase 1 and 2 clinical trials of the cPLA2 inhibitor, ZPL-521, showed that the drug was safe and well-tolerated even at high doses [231].

The action of thromboxane synthase on PGH_2_ produces 12-HHTrE (Fig. 3) (Gabbs et al., 2015), which is the main agonist of the BLT2 receptor [232], and whose activation strengthens the skin barrier by activating the p38 MAPK pathway, leading to the production of the adhesion protein claudin 4 (CLDN4) responsible for tight junctions between primary keratinocytes [233]. BLT2 receptor activation is also necessary for protection against epicutaneous sensitization and transepidermal water loss [233]. However, it is known that after skin damage, platelets produce large amounts of 12-HHTrE, which, by activating the BLT2 receptor in epidermal keratinocytes, accelerates wound healing by activating the NF-κB-TNF-α-MMPs metabolic pathway [234].

## Conclusions and future prospects

1. The integrity and, consequently, the proper metabolic functioning of skin cells are ensured by cell membranes, the basic structural and functional components of which are phospholipids, which are constantly enzymatically hydrolyzed to generate PUFAs, including arachidonic acid, which is a precursor of eicosanoids.
2. The main physiological function of eicosanoids is the constitutive control of normal and balanced cell proliferation, differentiation, and survival, as well as the regulation of skin cell inflammation.
3. Since dysregulation of eicosanoid levels may contribute to the development of skin diseases, this suggests that pharmacological regulation of eicosanoid generation, especially in psoriasis and atopic dermatitis (by: ZPL-521—cPLA2 inhibitor; zileuton—5-LOX inhibitor; timapiprant (NCT02002208)—DP2 receptor antagonist), as well as in the treatment of vitiligo (intradermal injection of PGE_2_ supported by NB-UVB phototherapy) is and will be developed.
4. For the purpose of using eicosanoids for identifying metabolic changes in skin disease and evaluating pharmacotherapy progress, further detailed studies are necessary to understand the relationship between specific eicosanoid and disease pathomechanism, as well as to develop an analytical approach for their diagnostic determination.

## Data Availability

Not applicable.

## Abbreviations

12-HHTrE: 12-Hydroxyheptadecatrienoic acid
15-d-PGJ_2_: 15-Deoxy-Δ12,14-PGJ2
20-COOH-LTB_4_: 20-Carboxy-LTB4
20-OH-LTB_4_: 20-Hydroxy-leukotriene B4
4-HNE: 4-Hydroxynonenal
6-keto PGF_1α_: 6-Ketoprostaglandin F1α
AA: Arachidonic acid
AAPC: Alkylacylglycerophosphocholines
ACD: Allergic contact dermatitis
AD: Atopic dermatitis
AGA: Androgenetic alopecia
AHR: Aryl hydrocarbon receptor
AP-1: Activator protein 1
AQP3: Aquaporin-3
AR: Androgen receptor
ARCI: Autosomal recessive congenital ichthyosis
AVX001: 1-Octadeca-2,6,9,12,15-pentaenylsulfanyl-propan-2-one
BAX: Bcl-2-associated X protein
Bcl-2: B-cell lymphoma 2
BLT1: Leukotriene B4 receptor 1
BLT2: Leukotriene B4 receptor 2
cAMP: Cyclic adenosine monophosphate
CCD 1112Sk: Human fibroblasts
CD206: Mannose receptor C type 1
CD25: CD25 T lymphocytes
CD4: CD4 T lymphocytes
Cer: Ceramides
CHS: Chronic hypersensitivity contact dermatitis
CL: Cardiolipins
CLDN4: Claudin 4
COXs: Cyclooxygenases
cPLA2: Cytosolic calcium dependent phospholipase A2
CREB: CAMP response element-binding protein
CRL2310: Human immortalized keratinocytes CDD 1102 KERTr
CXCL1: Chemokine (C-X-C motif) ligand 1
CXCL2: Chemokine (C-X-C motif) ligand 2
CXCR2: Motif chemokine receptor 2
cyPGs: Cyclopentenone prostaglandins
CYPs P450: Cytochromes P450
CysLT1: Cysteinyl leukotriene receptor 1
CysLT2: Cysteinyl leukotriene receptor 2
cys-LTs: Cysteinyl leukotrienes
DAG: 1,2-Diacylglycerol
DAGL: Diacylglycerol lipase
DCs: Dendritic cells
DHA: Docosahexaenoic acid
DHETs: Dihydroxyeicosatrienoic acids
DHS: Hydroxyeicosanoid dehydrogenases
DHSM: Dihydrosphingomyelins
DiHETEs: Dihydroxyeicosatetraenoic acids
DMBA/TPA: 9,10-Dimethylbenz(a)anthracene/12-O-tetradecanoylphorbol-13-acetate
DP1: Prostaglandin D2 receptor 1
DP2: Prostaglandin D2 receptor 2
e12-LOX: Epidermal-type 12-lipoxygenase
EETs: Epoxyeicosatrienoic acids
EGFR: Epidermal growth factor receptor
EHS: Epoxide hydrolases
EP1: Prostaglandin E2 receptor 1
EP2: Prostaglandin E2 receptor 2
EP3: Prostaglandin E2 receptor 3
EP4: Prostaglandin E2 receptor 4
EPA: Eicosapentaenoic *acid*
Epla: Plasmalogen ethanolamines
ERK1/2: Extracellular signal regulated kinase 1/2
EXA_4_: Eoxin A4
EXC_4_: Eoxin C4
EXD_4_: Eoxin D4
EXE_4_: Eoxin E4
FAAH: Fatty acid amide hydrolase
FP: Prostaglandin F receptor
GM22251: Primary human keratinocytes
GPL: Phosphoglycerides
GPx: Glutathione peroxidase
GSH: Glutathione
HaCaT: Human immortalized keratinocytes
hBD-3: β-Defensin 3
hDPCs: Human dermal papilla cells
HETEs: Hydroxy-eicosatetraenoic acids
HPEK: Human primary epidermal keratinocytes
HpETEs: Hydroperoxyeicosatetraenoic acids
HXA_3_: Hepoxilin A3
HXB_3_: Hepoxilin B3
ICAM1: Intercellular adhesion molecule 1
IL-1: Interleukin 1
IL-17A: Interleukin 17A
IL-17F: Interleukin 17F
IL-1β: Interleukin 1β
IL-22: Interleukin 22
IL-23: Interleukin 23
IL-31: Interleukin 31
IL-6: Interleukin 6
IMQ: Imiquimod
IP: Prostaglandin I2 receptor
IP3: Inositol-1,4,5-triphosphate
iPLA2: Cytosolic calcium independent phospholipase A2
IsoFS: Isofurans
IsoLG: Isolevuglandins
IsoP: Isoprostanes
IsoTX: Isothromboxanes
l12-LOX: Leukocyte-type 12lipoxygenase
LA: Linoleic acid
LC–MS/MS: Liquid chromatography with tandem mass spectrometry
LOXs: Lipoxygenases
LPA: Lysophosphatidic acid
LPC: Lysophosphatidylcholines
LPP: Lipid-phosphate phosphatase
LTA_4_: Leukotriene A4
LTA_4_H: LTA_4_ hydrolase
LTB_4_: Leukotriene B4
LTC_4_: Leukotriene C4
LTC_4_S: LTC_4_ synthase
LTD_4_: Leukotriene D4
LTE_4_: Leukotriene E4
LXA_4_: Lipoxin A4
LXB_4_: Lipoxin B4
MAG: Monoacylglycerol
MAGL: Monoacylglycerol lipase
MAPK: Mitogen-activated protein kinase
MDA: Malondialdehyde
MDCs: Macrophage*-*derived chemokine
MIP-2: Macrophage inflammatory protein 2
MMP-1: Extracellular matrix metalloproteinase 1
MMP-2: Extracellular matrix metalloproteinase 2
MPEK: Murine primary epidermal keratinocytes
MRSA: Methicillin-resistant Staphylococcus aureus
NAC: N-acetylcysteine
NAEs: N-acyl ethanolamines
NFAT: Nuclear factor of activated T cells
NHDF: Human dermal fibroblasts
NHEK: Normal human epidermal keratinocytes
NIH3T3: Murine fibroblasts
OSM: Oncostatin M
oxo-ETEs: Oxo-eicosatetraenoic acids
p12-LOX: Platelet-type 12-lipoxygenase
p38 MAPK: P38 mitogen-activated protein kinases
PA: Phosphatidic acid
p-Akt: Phosphorylated protein kinase B
PAM212: Murine keratinocytes
PAR-2: Proteinase-activated receptor 2
PC: Phosphatidylcholines
PE: Phosphatidylethanolamines
PG: Phosphatidylglycerols
PGA_2_: Prostaglandin A2
PGB_2_: Prostaglandin B2
PGD_2_: Prostaglandin D2
PGE_2_: Prostaglandin E2
PGG_2_: Prostaglandin G2
PGH_2_: Prostaglandin H2
PGI_2_: Prostaglandin I2
PGJ_2_: Prostaglandin J2
p-GSK3: Phosphorylated glycogen synthase kinase 3
PGXS: Prostaglandin synthase
PH: Pleckstrin homology
PI: Phosphatidylinositols
PIP2: 4,5-Bisphosphate phosphatidylinositol
PKA: Cyclic AMP-dependent protein kinase
PKCζ: Protein kinase C zeta
PLA1: Phospholipase A1
PLA2: Phospholipase A2
PLB: Phospholipase B
PLC: Phospholipase C
PLD: Phospholipase D
PLD1: Phospholipase D1
PLD2: Phospholipase D2
P-LPE: Plasmalogen-type lysoPE
PMK: Primary mouse keratinocytes
PPARs: Peroxisome proliferator-activated receptors
PPARγ: Peroxisome proliferator-activated receptor γ
PS: Phosphatidylserines
p-STAT3: Phosphorylated signal transducer and activator of transcription 3
PUFA: Polyunsaturated fatty acid
RANTES: Chemokine regulated upon activation normal T cell expressed and secreted
RhoA: Ras homolog family member A
ROS: Reactive oxygen species
RTK: Receptor tyrosine kinase
SM: Sphingomyelins
SP: Sphingolipids
sPLA2: Secretory phospholipase A2
TAG: Triacylglycerols
tetranor PGEM: 8-[(1R,2R,5R)-2-(2-carboxyethyl)-5-hydroxy-3-oxocyclopentyl]-6-oxooctanoic acid
TGF-β: Transforming growth factor beta
Th17: Lymphocytes Th17
Th22: Lymphocytes Th22
TNF-α: Tumor necrosis factor α
TP: Thromboxane receptor
TRPA1: Transient receptor potential cation channel, subfamily A, member 1
TRPV1: Transient receptor potential cation channel subfamily V member 1
TrXA_3_: Trioxilin A3
TrXB_3_: Trioxilin B3
TSLP: Thymic stromal lymphopoietin
TXA_2_: Thromboxane A2
TXB_2_: Thromboxane B2
TXS: Thromboxane synthase
UV: Ultraviolet
vLPR: Very late-phase
